# Effect of Chlorophyll Hybrid Nanopigments from Broccoli Waste on Thermomechanical and Colour Behaviour of Polyester-Based Bionanocomposites

**DOI:** 10.3390/polym12112508

**Published:** 2020-10-28

**Authors:** Bàrbara Micó-Vicent, Marina Ramos, Francesca Luzi, Franco Dominici, Valentín Viqueira, Luigi Torre, Alfonso Jiménez, Debora Puglia, María Carmen Garrigós

**Affiliations:** 1 Colour and Vision Group, University of Alicante, San Vicente del Raspeig, ES-03690 Alicante, Spain; barbara.mico@ua.es (B.M.-V.); valentin.viqueira@ua.es (V.V.); 2 Department of Appl. Stat. & Operat. Research, & Qual., Universitat Politècnica de València, ES-03801 Valencia, Spain; 3 Department of Analytical Chemistry, Nutrition & Food Sciences, University of Alicante, San Vicente del Raspeig, ES-03690 Alicante, Spain; marina.ramos@ua.es (M.R.); alfjimenez@ua.es (A.J.); 4 Department of Civil and Environmental Engineering, University of Perugia, 05100 Terni, Italy; francesca.luzi@unipg.it (F.L.); francodominici1@gmail.com (F.D.); luigi.torre@unipg.it (L.T.)

**Keywords:** broccoli waste, chlorophyll, hybrid nanopigment, experimental design, nanoclays, bionanocomposites

## Abstract

Natural dyes obtained from agro-food waste can be considered promising substitutes of synthetic dyes to be used in several applications. With this aim, in the present work, we studied the use of chlorophyll dye (CD) extracted from broccoli waste to obtain hybrid nanopigments based on calcined hydrotalcite (HT) and montmorillonite (MMT) nanoclays. The synthesized chlorophyll hybrid nanopigments (CDNPs), optimized by using statistical designed experiments, were melt-extruded with a polyester-based matrix (INZEA) at 7 wt% loading. Mechanical, thermal, structural, morphological and colour properties of the obtained bionanocomposites were evaluated. The obtained results evidenced that the maximum CD adsorption into HT was obtained when adding 5 wt% of surfactant (sodium dodecyl sulphate) without using any biomordant and coupling agent, while the optimal conditions for MMT were achieved without adding any of the studied modifiers. In both cases, an improvement in CD thermal stability was observed by its incorporation in the nanoclays, able to protect chlorophyll degradation. The addition of MMT to INZEA resulted in large ΔE* values compared to HT incorporation, showing bionanocomposite green/yellow tones as a consequence of the CDNPs addition. The results obtained by XRD and TEM revealed a partially intercalated/exfoliated structure for INZEA-based bionanocomposites, due to the presence of an inorganic filler in the formulation of the commercial product, which was also confirmed by TGA analysis. CDNPs showed a reinforcement effect due to the presence of the hybrid nanopigments and up to 26% improvement in Young’s modulus compared to neat INZEA. Finally, the incorporation of CDNPs induced a decrease in thermal stability as well as limited effect in the melting/crystallization behaviour of the INZEA matrix. The obtained results showed the potential use of green natural dyes from broccoli wastes, adsorbed into nanoclays, for the development of naturally coloured bionanocomposites.

## 1. Introduction

Natural dyes obtained from agro-food waste can be considered promising substitutes of synthetic dyes to be used in several applications [1], due to their easy availability, non-toxicity, antioxidant/antimicrobial activity, medicine applications and biodegradation capability showing no environmental issues [2,3,4]. In particular, broccoli wastes contain several nutrients and bioactive compounds such as pigments, glucosinolates and phenolic compounds, the valorisation of these wastes being very valuable for a wide range of industrial applications [5,6,7]. So, broccoli discards could be used as a potential source for the extraction of natural green dyes, including carotenoids (16–2689 µg g^−1^ DW) and chlorophylls (139–5569 µg g^−1^ DW), as already reported by Ferreira et al. [5]. However, natural dyes show some drawbacks such as challenging colour reproduction between different natural dye samples, low light fastness, restricted colour array, poor colour fastness and low chemical and thermal stability [8]. Chlorophyll dyes have been used in complex applications such as solar cell dyes due to their interesting properties [9,10], but the instability of these dyes under the exposure to oxygen, high temperature or light environments could limit their final applications [11,12].

Natural dyes are widely used in organic polymer formulations as colouring agents as well as to enhance other specific properties such as durability, mechanical properties and corrosion resistance. Different stabilization techniques such as microencapsulation and nanoclay adsorption are necessary to enhance the stability of natural dyes [13,14]. The use of modifiers, such as surfactants, silane agents and salts has been reported to improve nanoclay adsorption for different molecular dye structures [15,16,17,18,19]. Biomordant salts have been recently proposed as potential alternative additives to replace synthetic mordant salts in textile dyeing [20]. Hybrid dye-clay nanopigments have been reported to show good colour characteristics, excellent stability and mechanical performance [8,21,22]. Therefore, by combining the colour of natural dyes and the resistance of inorganic nanostructures, it is possible to obtain hybrid nanomaterials with improved chemical properties and higher stability [23]. The adsorption and intercalation mechanisms of reactive dyes within nanoclays for the development of nano-structured systems has been studied by different authors [24,25].

To the best of our knowledge, a minimal number of works have studied the synthesis of chlorophyll hybrid nanopigments [26] and their incorporation in polymer matrices. Specifically, while some authors investigated the role of these nanopigments in photodynamic therapy [27] and energy transfer in dye-sensitized solar cells [28], few authors investigated the effect of their introduction in polymer matrices. Ahmed et al. [29] showed that chlorophyll and anthocyanin could act as plasticizers affecting secondary bonds of polyvinyl chloride (PVC) polymer. In another work, Chandrappa et al. [30] successfully incorporated green tricolour leaf extract into a polyvinyl alcohol (PVA) matrix to obtain PVA-based biofunctional composite structures, showing the potential use of these biocomposites in UV shielding protective sheets/layers, windows or coatings for terrestrial and aquatic ecosystems. The smart response of chlorophyll, when combined with polypyrrole and gluten, was also proved by other authors, obtaining wheat gluten/chlorophyll/polypyrrole nanocomposite smart films due to their conductivity and colour change; additionally, chlorophyll pigment can enhance gluten film properties showing high potential for improving food shelf-life of packaged food [31,32]. In all these cases, dyes were introduced in their free state without using the hybrid combination with inorganic nanostructures.

The incorporation of hybrid nanopigments into polymer matrices could improve the physical and mechanical properties of the final nanocomposites [8,33,34]. Micó-Vicent et al. [19,22] demonstrated that the incorporation of hybrid nanopigments based on chlorophyll, beta-carotene and beetroot extract natural dyes into an epoxy bioresin improved the optical performance, thermal stability and UV-Vis light exposure stability of the natural dyes and bioresins. Mahmoodi et al. [35] reported the synthesis of hybrid dye-clay nanopigments and its application in epoxy coatings observing that the dyes chemically attached to the nanoclay particles do not migrate from the polymer matrix, and the resulting hybrid nanopigments showed better photo-stability and dispersion than that of pure dyes. The interesting properties of hybrid dye-clay nanopigments have been also applied in 3D printing technologies for the development of innovative coloured functional materials [36,37,38]. Mahmoodi et al. [39] also obtained coloured biodegradable/biocompatible poly(lactic acid) (PLA) nanocomposite films containing 1–5 wt% of a dye-clay hybrid nanopigment using a simple solution casting approach. High thermomechanical resistance as well as superior light and mass transport barriers for food packaging applications were observed for the developed nanocomposite films. Similar results were also obtained by Usopt et al. [40], who considered a dye containing chlorophyll from *Cassia alata* leaves as a colourant in blended poly(methylmetacrylate) (PMMA)-acrylic polyol for coating applications in the presence of copper (II) nitrate (Cu(NO_3_)_2_). The results from colour measurement showed that yellowness, glossiness and reflectivity were influenced by the natural dye, providing good colour stability in the produced films.

In this work, we propose the novel approach of synthesising chlorophyll hybrid nanopigments by using broccoli waste as a natural source and two nanoclay types with different ion exchange capacities. For this purpose, statistical experimental design experiments were considered to optimize the hybrid nanopigments preparation in the presence of different modifiers (surfactant, silane and natural biomordant obtained from pomegranate waste). We also verified, for the first time, how the incorporation of the produced chlorophyll hybrid nanopigments could enhance the overall behaviour (thermal, mechanical, morphological, structural and colour properties) of the bionanocomposites obtained by mixing the hybrid materials with a commercial biopolyester matrix.

## 2. Materials and Methods

### 2.1. Materials and Reagents

Broccoli waste, including leaves, stems and flowering parts, was obtained from discarded vegetables from FECOAM (Murcia, Spain). Broccoli waste was slightly cut into small pieces using a household blade cutter for 10 s at medium speed to reach particles of 1–50 mm^3^ [8].

Montmorillonite (MMT, Gel White) and hydrotalcite (HT, BioUltra, ≥99.0%) laminar nanoclays, with a different charge ion capacity, were supplied by Southern Clay Products (Gonzales, TX, USA) and Sigma-Aldrich (St. Louis, MO, USA), respectively. HT was calcined at 600 °C for 3 h before use. Cetylpyridinium bromide (CPB) and sodium dodecyl sulphate (SDS) were used as surfactants for MMT and HT, respectively. A coupling agent (3-Aminopropyl) triethoxysilane (as silane) and a natural biomordant obtained from pomegranate waste peel (FECOAM, Murcia, Spain) were used as surface modifiers. All reagents and chemicals were of analytical grade, and they were purchased from Sigma-Aldrich (St. Louis, MO, USA).

INZEAF2 biopolyester commercial grade (density of 1.23 g cm^−3^ at 23 °C; moisture content < 0.5 %; melt flow rate of 19 g 10 min^−1^ (2.16 kg, 190 °C) was kindly provided by Nurel (Zaragoza, Spain) and used for bionanocomposites preparation.

### 2.2. Broccoli Dye and Pomegranate Peels Biomordant Extraction

A FLEXIWAVE™ microwave oven (Milestone srl, Bergamo, Italy) was used to obtain the natural additives used in this work, chlorophyll dye (CD) from broccoli wastes and pomegranate biomordant, by following methods previously optimized.

For CD, 20 g of freshly cut broccoli waste and 90% (*v*/*v*) acetone were used for microwave-assisted extraction (MAE) at a liquid to solid ratio of 5:1 in a 250 mL round-bottom flask. The sample was heated at 6 W g^−1^ for 25 min under reflux and stirring (400 rpm). The boiling temperature was reached at around 60 °C and maintained during extraction. After MAE, 5 mL of water containing 5 wt% of sodium carbonate (Na_2_CO_3_) was used to maintain the stability of the extracted chlorophylls.

To obtain the biomordant from pomegranate waste, peels were dried at 40 °C for 24 h in a climatic chamber (Dycometal, Barcelona, Spain) at a relative humidity (RH) of 25%. Dried peels were grounded with a high-speed rotor mill at 12,000 rpm (Ultra Centrifugal Mill ZM 200, RETSCH, Haan, Germany). Particles passing through a 0.5 mm sieve were used. MAE was performed by using 6 g of pomegranate sample mixed with 60 mL of 40% (*v*/*v*) ethanol (solid to solvent ratio of 1:10) for 10 min at 65 °C.

The obtained CD and biomordant extracts were filtered, and polysaccharide compounds were precipitated by adding 96% (*v*/*v*) ethanol. Samples were kept overnight at −20 °C to promote precipitation of insoluble compounds, and they were vacuum-filtered. The solvent present in the samples was firstly removed in a rotary evaporator (R-300, Büchi Labortechnik AG, Switzerland) and the aqueous solution was freeze-dried (LyoQuest Plus, Telstar, Terrassa, Spain). The purified additives were stored in the darkness at room temperature until further use.

### 2.3. Synthesis of Chlorophyll Hybrid Nanopigments (CDNPs)

The synthesis of the chlorophyll hybrid nanopigments (CDNPs) was performed by following the water/organic solvent dispersion method, based on previous studies [19]. A 2^4–1^ fractional factorial design of experiments (DoE) consisting of 8 experiments was used to study the best synthesis conditions to maximize broccoli dye adsorption into the laminar nanoclays. The solvent used in all experiments was 50 % (*v*/*v*) ethanol due to the low solubility of chlorophyll dye shown in water. The total amount of dye loaded for each sample was 0.2 g in 3 g of nanoclay. Four independent variables at two levels were considered (Table 1): nanoclay ion exchange capacity (HT or MMT), surfactant concentration (0 and 5 wt%), biomordant concentration (0 and 1 wt%) and silane concentration (0 and 5 wt%). The amount of intercalated dye in the nanoclay system was determined using a UV-Vis spectrophotometer (JASCO V650, Easton, MD, USA) at 405 nm by calculating the dye concentration present in the separated supernatants. Calibration curves were prepared in 50% (*v*/*v*) ethanol. The dye adsorbed over the initially added dye (%) was used as response to be maximized in the DoE analysis.

In addition, the final visual appearance of the optimal CDNPs and their thermal properties were evaluated using thermogravimetric analysis (see conditions in Section 2.5).

### 2.4. Bionanocomposites Preparation

Bionanocomposites based on INZEAF2 were obtained by melt blending the biopolymer and the synthetized CDNPs at 7 wt%. A co-rotating twin-screw extruder, Xplore 5 and 15 Micro Compounder by DSM, was used by mixing at a rotating speed of 90 rpm for 3 min, setting a temperature profile of 190–195–200 °C in the three heating zones from the feeding section to die. A Micro Injection Moulding Machine 10cc by DSM, coupled to the extruder and equipped with adequate moulds, was used to produce samples for tensile tests according to the standards. An appropriate pressure/time profile was used for the injection of each type of samples, while the temperatures of the injection barrel and the moulds were set, respectively, at 210 and 30 °C.

### 2.5. Bionanocomposite Characterization

Thermal degradation of CD and CDNPs as well as bionanocomposites was evaluated by thermogravimetric analysis (TGA, Seiko Exstar 6300, Tokyo, Japan). Around 5 mg of samples was heated from 30 to 800 °C at 10 °C min^−1^ under nitrogen atmosphere (200 mL min^−1^). Three replicates of each sample were performed.

Differential scanning calorimetry (DSC) tests for bionanocomposites were conducted, in triplicate, using a DSC Q200 (TA Instruments, New castle, DE, USA) under nitrogen atmosphere (50 mL min^−1^). Samples of 3 mg were introduced in aluminum pans (40 µL), and they were submitted to the following thermal program: −30 to 250 °C at 10 °C min^−1^, with two heating scans and one cooling scan.

Dynamic mechanical thermal analysis (DMTA) of produced materials was performed, in triplicate, by using an Ares N2 rheometer (Rheometric Scientific, Epsom, Surrey, UK). Samples with dimensions of 2 × 5 × 40 mm, gripped with a gap of 20 mm, were tested in rectangular torsion at a frequency of 2π rad s^−1^ with a strain of 0.05%. A temperature ramp of 3 °C min^−1^ was applied in the range from 30 to 110 °C.

Optical properties of bionanocomposites were studied with a Konica Minolta sphere integrated spectrophotometer (CM-2300d, Tokyo, Japan). Data were acquired by using the SCI 10/D65 method, whereas CIELAB colour variables were used. Samples were placed on a white standard plate and L*, a* and b* parameters were determined. Measurements were performed, in triplicate, at random locations on each sample. Total colour difference ΔE_ab_* was calculated with the obtained colorimetric attributes of the CIELAB colour space.

Tensile tests were carried out for bionanocomposites by using a universal test machine LK30 (Lloyd Instruments Ltd.) at room temperature according to ASTM D638-14 Standard. A minimum of five different samples was tested using a 5 kN load cell, setting the crosshead speed to 5 mm min^−1^.

The CDNPs dispersion in the bionanocomposites was studied by transmission electron microscopy (TEM) and wide angle X-ray scattering (WAXS). TEM micrographs were obtained with a JEOL JEM-2010 (Tokyo, Japan) with an accelerating voltage of 100 kV. WAXS patterns were recorded at room temperature using a Bruker D8-Advance diffractometer (Madison, WI, USA) at scattering angles (2θ) 2.5°–80° (scanning rate of 3 s step^−1^ and step size of 5°) using filtered Cu Kα radiation (1.54 Å).

### 2.6. Statistical Analysis

Statistical analysis of results was performed by using Statgraphics Centurion XVI (Statistical Graphics, Rockville, MD, USA) to generate and analyse the results of the experimental design. The graphic analysis of the main effects and interactions between variables was used and the analysis of variance (ANOVA) was carried out. Differences between average values were assessed based on the Tukey test at a confidence level of 95% (*p* < 0.05). Characterization results for bionanocomposites were expressed as the mean ± standard deviation.

## 3. Results

### 3.1. Chlorophyll Hybrid Nanopigments (CDNPs)

The synthesis conditions for CDNPs were optimized by calculating the dye adsorption (Ads, %) as the concentration difference between the initially added dye in the nanoclay dispersion and the dye separated from the solvent after centrifugation. As can be seen in Table 1, good values for dye adsorption were obtained ranging from 95.66 to 98.44%, indicating a good degree of incorporation of the chlorophyll dye (CD) into HT and MMT nanoclays.

**Figure 1:**
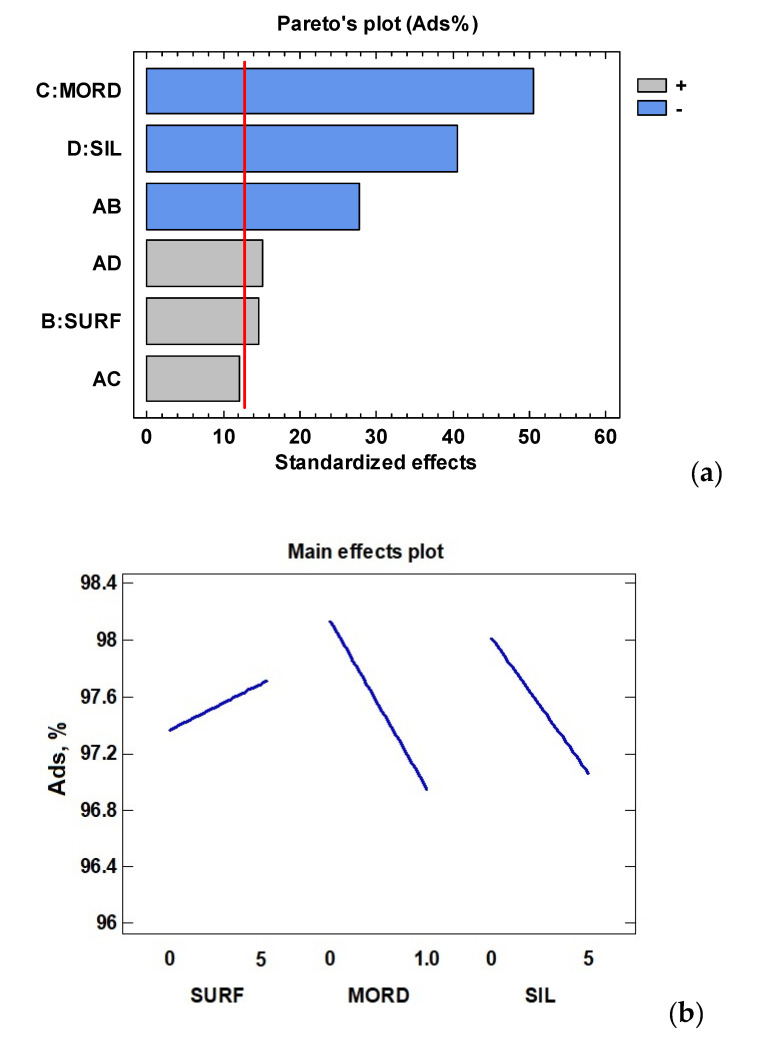
Pareto (**a**) and main effect (**b**) plots for MMT and HT-based chlorophyll hybrid nanopigments.

Analysis of variance (ANOVA) was performed to evaluate the effect of the studied variables on the dye adsorption (Table 2). A high degree of correlation between experimental and predicted values was obtained (R^2^ = 99.98%). The statistical analysis showed that the synthesis of CDNPs was mainly influenced by the biomordant and silane modifiers (with a negative effect), followed by the surfactant which a positive effect—as can be seen in the Pareto and main effect plots (Figure 1).

However, the nanoclay structure used for CD adsorption (HT or MMT) was not found to be significant (*p* > 0.05), and just slightly higher adsorption values were obtained for HT compared to MMT (Table 1). So, the interactions shown between the used modifiers and the nanoclay (Figure 1) should be considered to explain interlayer and surface modifications of the used nanoclays that could favour CD adsorption [41]. The aggregation and molecular orientation of the natural dye and layer charge density are also determinant parameters to be considered in the synthesis process of dye/clay complexes [42]. Additionally, the molecular weight and structure of the natural dyes have been reported to directly affect the dye adsorption process on the absorbent surface. In this sense, lower molecular weight dyes could present a higher mobility during the adsorption process and consequently lead to a higher adsorption rate [43]. In our case, the molecular weight of CD could be difficult to determine, to some extent, due its incorporation into both HT and MMT nanoclays.

**Figure 2:**
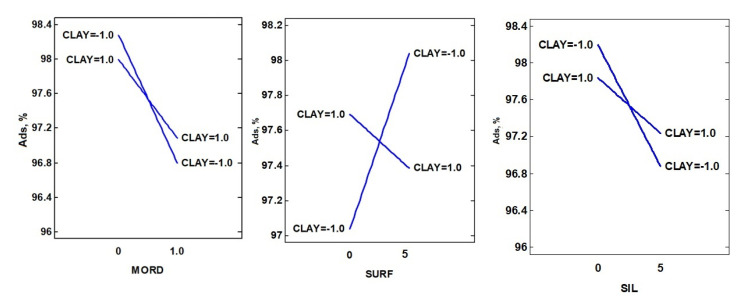
Interaction plots for the synthesis optimization of chlorophyll hybrid nanopigments. For nanoclay codification, (−1) corresponds to HT and (+1) to MMT.

Strong significant (*p* < 0.05) interactions between nanoclay-surfactant (negative) and nanoclay-silane (positive) were observed (Figure 2). A positive (*p* > 0.05) interaction between nanoclay-biomordant was also found. The influence of the nanoclay structure and the combined addition of silane, mordant and surfactant modifiers in the nanopigment synthesis of different natural dyes has been previously studied [22]. In this work, the use of the surfactant (SDS) increased the adsorption of the dye into HT, while, in contrast, the presence of CPB retained, to a higher extent, the amount of CD loaded into the clay structure for MMT. Regarding the use of the biomordant and silane, a similar trend was found as the adsorption of CD favoured in the absence of both modifiers. So, it was concluded that for MMT-based CDNPs, it was better not to use any of the selected modifiers, while for HT-based nanopigments, just the addition of the surfactant (CPB) will be needed to increase the CD content adsorbed.

**Figure 3:**
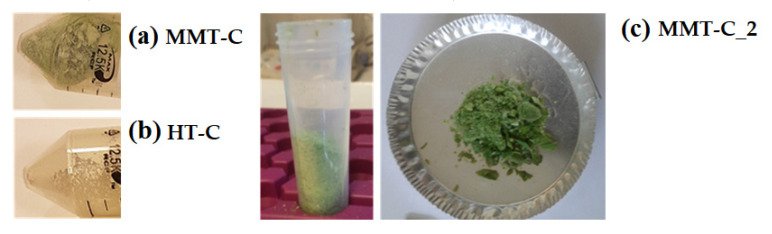
Visual appearance of chlorophyll hybrid nanopigments obtained with MMT (MMT-C) (**a**), HT (HT-C) (**b**) and MMT with a higher chlorophyll dye (CD) concentration (MMT-C_2) (**c**).

Figure 3a,b show the final appearance and colour of the optimized CDNPs (MMT-C and HT-C, containing, respectively, 0.2 g of chlorophyll dye in 3 g of MMT and HT). In order to obtain a more greenish intense colour, a new sample containing 1 g of chlorophyll dye in 3 g of MMT, without the addition of any modifier, was also obtained (MMT-C_2, Figure 3c).

#### Thermal Stability of CDNPs

**Figure 4:**
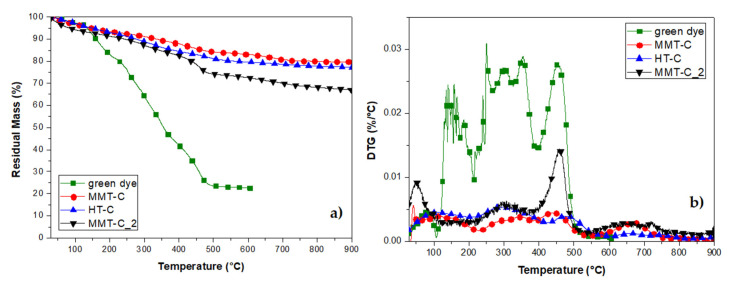
TG (**a**) and DTG (**b**) results obtained for CD (green dye) and chlorophyll hybrid nanopigments (MMT-C, HT-C and MMT-C_2).

The obtained nanopigments (MMT-C, HT-C and MMT-C_2) were also thermally characterized by TGA analysis, and the obtained results are shown in Figure 4. The weight loss profile and the derivative curve of the extracted green chlorophyll dye (CD), without being incorporated in the nanoclays, was also studied and included in the TG/DTG curves. The stability of chlorophyll in plants is known to be affected by a number of factors such as moisture (water), oxygen, temperature, light, pH, long time storage and the presence of enzymes or metals; chlorophyll is easily degraded in the presence of any of these factors resulting in pheophytin as a degradation product in a primary stage. According to Samide and Tutunaru [44], the first step of mass loss in CD observed up to 100 °C was attributed to the removal of physically adsorbed substances on the dye surface during extraction. The chlorophyll transformation reaction continues in the temperature range 100–150 °C, where the decomposition of pheophytin to pyropheophytin takes place. In a next step, the pyropheophytin decomposition to pyropheophorbide began and this step continued up to 500 °C, where the loss was stabilized obtaining a final residue of nearly 20 wt% attributed to carbonaceous magnesium oxide (Figure 4).

The low thermal stability observed for CD was improved by its incorporation in the nanoclays, protecting chlorophyll degradation to some extent under thermal processing conditions (Figure 4). According to reported results and previous observation of unmodified nanoclays, the first step observed in the TG curves of the hybrid nanopigments up to 200 °C was attributed to the desorption of water molecules between the layers, for both MMT and HT; while the further weight loss above 400 °C was ascribed to the dihydroxylation of the remaining OH groups of the nanoclays [8]. The thermal profiles observed for MMT-C and HT-C nanopigments were quite similar, showing a final residual mass of nearly 80 wt% in both dye/clay systems. In addition, the main peaks observed for mass loss in their DTG curves were located at the same temperature range as that observed for CD degradation. On the other hand, the MMT-C_2 nanopigment, with a more brilliant and intense green colour, showed a more evident weight loss and lower thermal stability, essentially due to a higher CD loading on MMT clay, showing a superposition of the thermodegradative behaviour of both MMT and CD components.

### 3.2. Bionanocomposites Characterization

**Figure 5:**
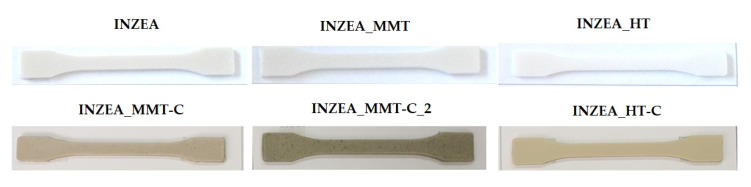
INZEA-based bionanocomposites containing unmodified nanoclays and chlorophyll hybrid nanopigments at 7 wt%.

The produced CDNPs were incorporated at 7 wt% loading for the development and characterization of INZEAF2-based coloured bionanocomposites. Blank samples corresponding to neat INZEA and INZEA containing unmodified MMT and HT at 7 wt% were also obtained. Figure 5 shows the visual images of all obtained biobionanocomposites. A darker green colour was observed for the INZEA_MMT-C_2 sample due to the intrinsic colour of MMT and MMT-C_2 nanopigment which contained a higher CD amount.

#### 3.2.1. Evaluation of Colour Parameters

The results obtained for the colour parameters of the developed bionanocomposites are included in Table 3**.** Neat INZEA was characterized by a high lightness value due to the colour and clear appearance of the neat polymer. The addition of MMT and HT to INZEA-based systems produced, respectively, a reduction and increase in L* values (Table 1). The addition of MMT to INZEA resulted in large ΔE* values compared to HT incorporation, with values of 21.89 and 13.93, respectively. This phenomenon was associated to the MMT and HT intrinsic colours, which were able to modify the final aesthetic quality and appearance of the different samples [8]. The presence of CDNPs in INZEA-based systems determined a remarkable variation in CIELAB values. A visible reduction in L* and a* values and an increase in b* values were observed, compared to the unmodified clays, indicating a deviation of the polymer samples towards green/yellow tones and an overall decrease in the sample lightness. The lower lightness in those samples containing chlorophyll hybrid nanopigments could be attributed to their selective light absorption and green colour which could hinder light transmittance [39]. Regarding MMT-based chlorophyll hybrid nanopigments, the MMT-C sample was characterized by a bright green colour compared to INZEA_MMT-C_2, showing a higher ΔE* value in the latter. Finally, gloss values were reduced by using unmodified nanoclays and partially recovered with the incorporation of CDNPs, which contributed to a great extent to modifying the ΔE* values.

#### 3.2.2. Tensile Properties

**Figure 6:**
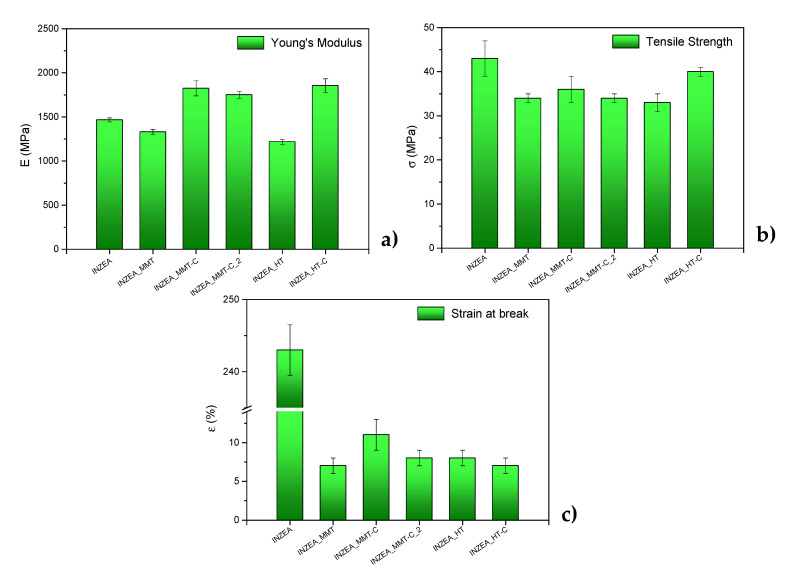
Results of tensile tests for INZEA-based bionanocomposites containing 7 wt% of unmodified MMT and HT and chlorophyll hybrid nanopigments: (**a**) E, Young’s Modulus; (**b**), σ_b_: strength at break and (**c**) ε_b_ at σ_b_: strain at break. (m ± SD; n = 5).

Tensile characterization results of INZEA-based bionanocomposites are reported in Figure 6. The incorporation of 7 wt% of CDNPs resulted in an increase in Young’s modulus of 24.4% and 26.5% for INZEA_MMT-C and INZEA_HT-C, respectively, demonstrating a reinforcement effect of the developed nanopigments in the INZEA matrix. The mechanical properties of the nanocomposites greatly depend on the interaction between the matrix components (polymer and nanoclay). The formation of intercalated/exfoliated structures could enhance the interfacial interactions between the polymer molecular chains and nanopigment layers, resulting in higher stiffness [39]. However, the increase in CD loading in INZEA_MMT-C_2 produced some deterioration in E values compared to INZEA_MMT-C. Marchante et al. [33] also described a similar trend in the Young’s modulus when nanoclay-based pigments were incorporated into linear low-density polyethylene. On the other hand, a decrease in terms of strength and strain at break was observed for all formulations, regardless of the type of the nanopigment introduced in the polymer matrix. Similar findings were reported by Esfahani et al. [45] which were attributed to the existence of weak polymer/nanoclay interactions and a decrease in ductility. The formation of some agglomeration of nanopigment particles and the presence of unexfoliated aggregates and structural voids could result in a limited amount of intercalation/exfoliation of MMT and HT in the polymer matrix, affecting the structural stability and mechanical performance of the final bionanocomposites and explaining the observed mechanical results [46].

#### 3.2.3. DMTA Tests

**Figure 7:**
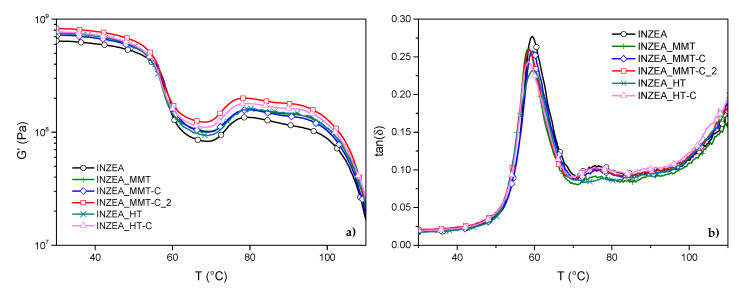
G’ (**a**) and tan δ curves (**b**) for INZEA-based bionanocomposites containing 7 wt% of unmodified MMT and HT and chlorophyll hybrid nanopigments.

Dynamic mechanical thermal analysis was considered to further evaluate the effect of the addition of the CDNPs on the thermomechanical behaviour of the neat INZEA matrix. The analysis of G’ curves (Figure 7a) revealed that while the unmodified MMT and HT incorporation slightly improved the original INZEA values (7.1 × 10^6^ MPa and 7.0 × 10^6^ MPa, respectively, for INZEA_MMT and INZEA_HT compared to 6.1 × 10^6^ MPa for neat INZEA), the storage moduli for the formulations containing hybrid nanofillers were enhanced with respect to the neat matrix. The adsorption of the CDNPs onto the macromolecular chains of the biopolyester led to a constraint in the chains movement, particularly in the case of MMT-based hybrid nanopigments. In particular, after the incorporation of 7 wt% of MMT-C and MMT-C_2, the storage modulus (measured at 40 °C) of neat INZEA increased up to 7.1 × 10^6^ MPa and 7.8 × 10^6^ MPa, respectively for INZEA_MMT-C and INZEA_MMT-C_2, demonstrating the reinforcing effect of high loaded CDNPs in the polyester matrix. The possible formation of an intercalated/exfoliated morphology (as demonstrated in the following section) enhanced the interfacial interactions between the polymer molecular chains and the nanohybrid layers. Consequently, the imposed stress on polymer chains can be effectively suppressed by the rigid and high aspect ratio clay nanosheets of CDNPs [39]. The formation of some aggregates and a decrease in the amount of intercalation/exfoliation of CD in the case of HT-based nanohybrids can explain the observed results in terms of G’ values for these hybrid nanopigments, registering storage moduli (measured at 40 °C) of 7.0 × 10^6^ MPa and 7.3 × 10^6^ MPa, respectively, for INZEA_HT and INZEA_HT-C. The upper temperature limit of application of these materials is close to the melting point of the low melting component—i.e., 100 °C—above which the modulus decreases rapidly.

Regarding the determination of the *T*_g_ by DMTA (Figure 7b), a signal related to the presence of a glass transition at around 60 °C was monitored and ascribable to the transition of the high melting fraction; in parallel, a slight variation in glass transition temperatures after the incorporation of the nanofillers for the other samples was noted, meaning that a restriction in the movement of the polyester macromolecular chains occurred, significantly affecting the stiffness of the materials. These results may be explained by the fact that the dispersed nanoclay layers in the polyester matrix can decrease the free volume and hinder the segmental motions of PLA molecular chains at the interface, thus leading to an increase in glass transition temperature [47]. Additionally, a drop in the intensity of the tan δ peak was observed for bionanocomposite samples which was amplified in the presence of CDNPs compared to neat INZEA and filled INZEA with MMT and HT. In this sense, the embedded high aspect ratio CDNP layers in the INZEA matrix could cause a strong interaction between the two components, restricting the polymer chain motions.

#### 3.2.4. Structural and Morphological Properties

**Figure 8:**
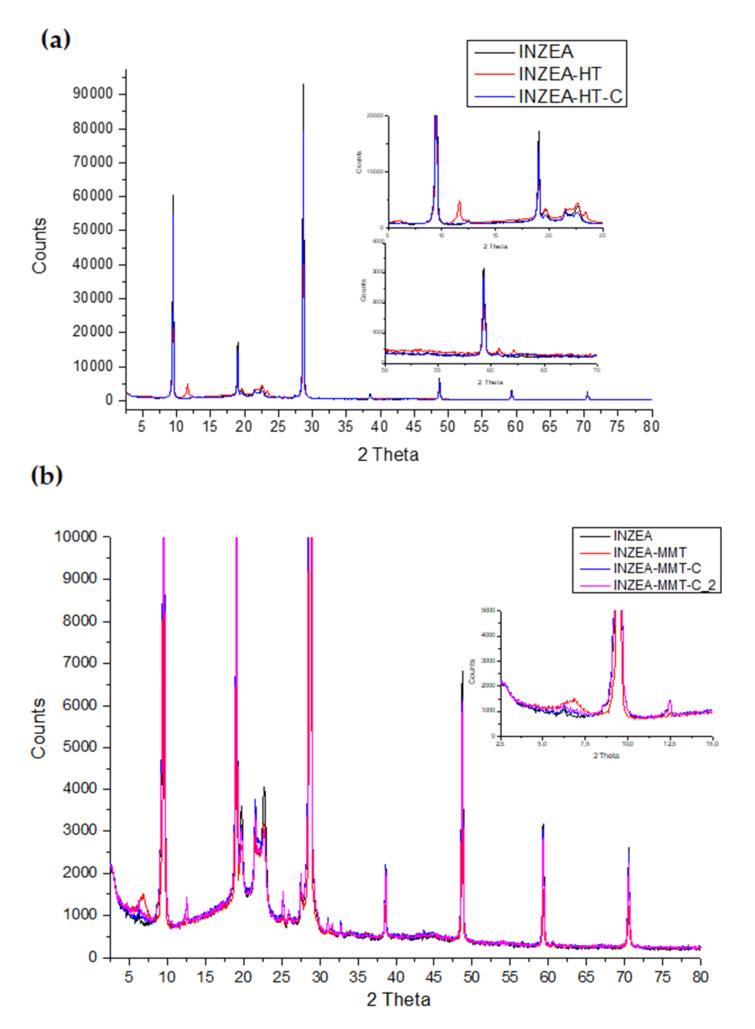
XRD patterns of (**a**) INZEA and INZEA-based bionanocomposites containing 7 wt% of unmodified HT and chlorophyll hybrid nanopigment; (**b**) INZEA and INZEA samples containing 7 wt% of unmodified MMT and chlorophyll hybrid nanopigments.

**Figure 9:**
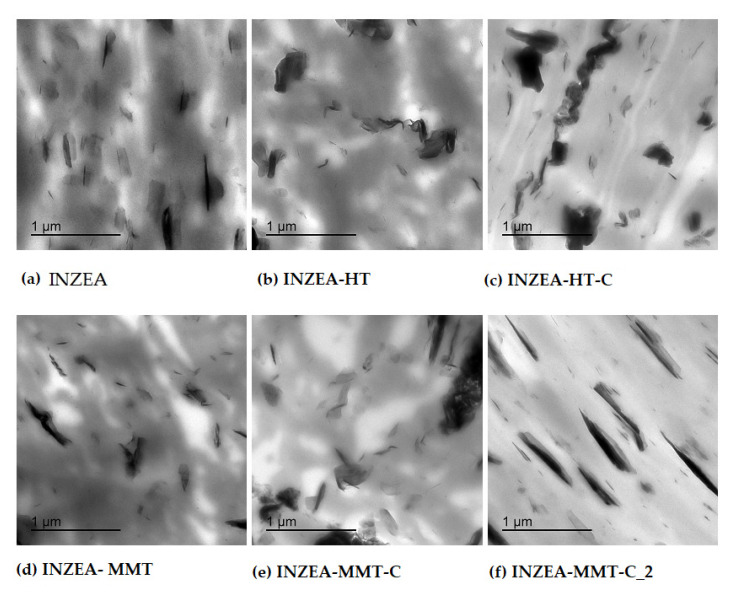
TEM micrographs of INZEA-based bionanocomposites containing 7 wt% of unmodified MMT and HT and chlorophyll hybrid nanopigments.

Figure 8a shows the XRD patterns of INZEA, INZEA-HT and INZEA-HT-C materials. As can be observed, INZEA showed two sharp and intense peaks at 9.5° and 28.6° that could be attributed to an inorganic filler present in the polymer blend structure. Some authors have attributed these peaks to the crystallographic pattern of talc, in which tetrahedral sheets constituted by a network of silicon and oxygen atoms surrounded by Mg(OH)_2_ sheets are the main constituents of this mineral reinforcement [48,49]. This result is also in agreement with the TEM micrographs shown in Figure 9 giving a clear indication of the presence of an amount of an inorganic filler in the INZEA structure. The relatively low-intensity peaks around 19–22° match the diffraction of poly(butylene succinate) (PBS), particularly the peaks at 2θ = 19.4° and 22.2°, corresponding to the (020) and (110) crystallographic planes, respectively [50]. Another low-intensity peak was observed at around 16.7°, and it could be attributed to the PLA fraction of the polymer blend, in particular the (200) crystallographic plane, as has already been reported by other authors [51].

When HT nanoclay was added to the INZEA matrix, two low-intensity but relevant peaks at around 2θ = 11.6° and 23.4° (see red curve in zoomed area in Figure 8a) were observed. These peaks are indicative of the successful incorporation of this nanoclay into the INZEA structure, and they can be attributed to the (003) and (006) crystallographic planes of calcined HT modified with the addition of a surfactant (SDS), as has already been reported [17,19]. Two other peaks were observed at high angles between 62° and 63° which were assigned to the (110) and (113) crystallographic planes of the surfactant-modified HT, according to previous reported works [19,52,53]. All these peaks assigned to HT were not observed with the incorporation of the chlorophyll hybrid nanopigment (INZEA-HT-C), giving an indication of a modification in the HT crystalline structure and a partial intercalation/exfoliation of CD into the HT laminar structure, resulting in the modification of the HT planes as reported in other formulations [52,53] and in agreement with TEM results (Figure 9).

Figure 8b shows the XRD patterns of INZEA-based formulations modified with MMT and CDNPs at different CD concentrations. The presence of the characteristic crystallographic peak of MMT (around 7°) was confirmed (see red curve in the zoomed area in Figure 8b), corresponding to the (001) plane [19]. The observation of this peak has been extensively reported as indicative of the successful incorporation of MMT into different biopolymer blends, such as those based on PLA and poly(butylene succinate)-co-adipate (PBSA) [54]. However, this peak was not observed when CD was incorporated into the MMT structure giving rise to similar conclusions to the case of HT-based nanocomposites, suggesting a partial intercalation/exfoliation of CD into the MMT laminar structure and the modification of the nanoclay structure with the loss of its crystalline character.

As already stated, the obtained XRD results were in agreement with the TEM micrographs shown in Figure 9. A surface study was performed to precisely evaluate the incorporation and dispersion of CDNPs into the polymer blend structure. The micrograph obtained for INZEA (Figure 9a) showed a random dispersion of tactoids with different sizes all distributed through the polymer matrix. This is another clear indication of the presence of an inorganic filler in the polymer blend structure. The combined XRD and TEM results found for neat INZEA were in agreement with those recently reported by Dias et al. [55], who did not observe a good dispersion and just some homogeneous distribution of the inorganic filler in polyurethane-based nanocomposites. These authors also reported that talc tactoids in these formulations were around 500 nm wide, which are similar to the results obtained in this study.

The addition of hydrotalcite, with or without CD, to the INZEA matrix resulted in the modification of the polymer microstructure, since some agglomeration was observed probably caused by the interaction between both nanofillers (the intrinsic inorganic filler present in the INZEA structure and HT nanoclay), with some indications of a partial intercalation/exfoliation, particularly when CD was present (Figure 9b,c). These results could be explained when considering the anionic character of the surfactant used, since its addition into the HT together with CD would result in the delamination of the nanoclay platelets and the consequent modification of the nanocomposite structure, as observed for other anionic dyes [56]. A similar behaviour was observed for MMT-based bionanocomposites (Figure 9d–f), where the presence of partially intercalated/exfoliated structures after the addition of MMT-based CDNPs was obtained.

However, it should be highlighted that these results were slightly different from those expected, since the intercalation of the used nanoclays, with or without CD addition, should be higher, according to previous results reported for PLA/PBS blends added with organo-modified MMT where a high intercalation/exfoliation degree was reported [57]. The mechanism of incorporation of the nanopigments and the nanoclay itself into the polymer matrix could be clearly interfered by the presence of another filler, as already discussed, making the possibilities for intercalation difficult. These observations suggest that the intercalation of the unmodified MMT and HT and CDNPs into the INZEA structure was more complicated and complex than expected due to the presence of grafted tactoid-like structures in the polymer matrix, as observed with other polymer matrices reinforced with MMT [58]. This effect could also explain the results obtained in the tensile tests which were described in Section 3.2.2.

#### 3.2.5. Thermal stability

**Figure 10:**
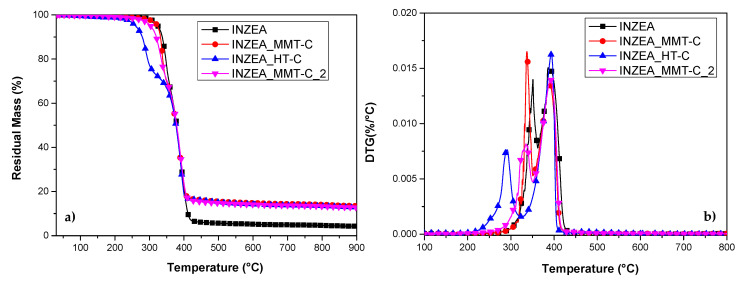
TG (**a**) and DTG (**b**) profiles obtained for INZEA-based bionanocomposites containing 7 wt% of functionalized MMT and HT with CD.

A deep analysis of the DTG curves (Figure 10) obtained for bionanocomposites containing HT-based CDNPs clearly indicated that the thermal stability of the overall blend was strongly affected. Specifically, while the temperature for the second main peak remained substantially unaffected, the T_peak1_ for the less stable component in the INZEA matrix was shifted from 350 to 291 °C for INZEA_HT-C. An analogous behaviour, even if limited in the shift values, was noted for MMT-based materials, where T_peak1_ for the less stable component (registered at 352 °C) shifted to 346 and 337 °C for INZEA_MMT-C and INZEA_MMT-C_2, respectively. According to the literature, this degradation path can be rationalized by considering the organic nature of the nanopigments, which were indeed responsible for the decrease in thermal stability of the polyester-based matrix, due to a possible hydrolysis reaction [59,60]. On the other hand, a residual mass for the unfilled matrix of 4 wt% was observed at 900 °C indicating the presence of an inorganic filler in the formulation of the commercial product, as already reported in a previous study [61] and observed in XRD and TEM results. The incorporation of CDNPs into the polymer matrix limited the movement of the polymer phase, thereby reducing the residual weight loss of the bionanocomposite materials, with a mean increase of 13 wt%. It is well known that only when nanoclay layers are exfoliated, they may have reduced the volatilization of the degradation products, and then, the thermal stability could increase [62]. In our case, the already observed limited mechanical enhancement, due to reduced exfoliation of the nanoclays, can also justify the reduced thermal stability to some extent in the obtained formulations.

**Figure 11:**
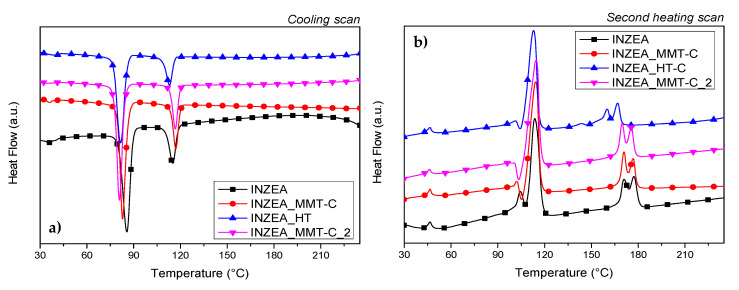
Curves of DSC cooling (**a**) and second heating (**b**) scans performed at 10 °C min^−1^ for INZEA-based bionanocomposites containing 7 wt% of functionalized MMT and HT with CD.

Figure 11a,b show the DSC thermograms obtained for bionanocomposites during the cooling and second heating scans. Two different peaks were observed for neat INZEA, indicating the presence of the two main polyesters in the polymer matrix, in agreement with the behaviour previously observed in a previous work [8]. While similar values for glass transition temperatures were registered, a significant effect due to the presence of CDNPs on the melting/crystallization behaviour of the polymer matrix was detected. A different trend was noted in the shift of T_c_ temperatures in the case of low and high melting fractions, and at the same time MMT- and HT-based nanopigments induced a different crystallization effect (Figure 11a). In the case of MMT-based formulations (INZEA_MMT-C and INZEA_MMT-C_2), a slight shift towards higher melting temperatures (from 115 °C for the neat polymer to 117 °C for bionanocomposites) was observed, while the crystallization was indeed delayed observing a peak shift to lower temperatures (from 86 °C for neat INZEA to 83 and 81 °C, respectively, for INZEA_MMT-C and INZEA_MMT-C_2). By considering the possible composition of the commercial INZEA as a blend of two polyesters, a better dispersion for the MMT-based CDNPs in the lower melting phase was suggested, according to the observed melting curves (second scan, Figure 11b), which registered small melting variations only for this fraction. On the other hand, it was found that the presence of nanopigments intercalated in HT altered the bimodal melting behaviour of the high melting fraction, and it is expected that a concurrent effect occurred, for a limited nucleating action and partial hydrolysis of the polyester agent due to the presence of hydroxyl surface functionalities for HT [63].

## 4. Conclusions

Coloured reinforced INZEA-based bionanocomposites containing 7 wt % of chlorophyll hybrid nanopigments (CDNPs) were developed by melt blending. CDNPs were successfully synthetized by using a chlorophyll dye (CD) obtained from broccoli wastes and two nanoclays, MMT and calcined HT. The best synthesis conditions for CD adsorption were studied by using a 2^4–1^ fractional factorial design of experiments. The solvent used was 50% (*v*/*v*) ethanol, and the total amount of dye loaded was 0.2 g in 3 g of nanoclay. The incorporation of CD into the nanoclays improved its thermal stability. The maximum CD adsorption into MMT was obtained without the addition of any modifier, while 5 wt% of surfactant (sodium dodecyl sulphate) was needed in the case of HT. Interesting green/yellow tones were obtained with the addition of CDNPs to the INZEA polyester matrix. The morphological and structural XRD characterization of INZEA-based bionanocomposites showed the coexistence of an inorganic filler in the commercial formulation together with the hybrid nanofillers, affecting the agglomeration and intercalation/exfoliation state of CDNPs in the polymer matrix. For the same reason, the incorporation of CDNPs into the polymer matrix was found to affect the thermal stability of the bionanocomposites, by reducing, to some extent, the temperature of maximum degradation rate for the less thermally stable component in the INZEA matrix. The melting/crystallization behaviour of the polyester-based matrix was also modified by the presence of the CDNPs, since the presence of the nanopigments altered the bimodal melting behaviour of the high melting fraction and delayed the crystallization towards lower temperatures.

The developed coloured polyester-based bionanocomposites could be considered as promising functional materials to be potentially used in different applications where desired colour and reinforcement properties could be an issue, such as in the automotive sector. In addition, the use of chlorophyll dyes obtained from broccoli wastes could contribute to the valorisation of these residues and the general objective of the circular economy approach. Further work will be needed to improve the interaction between the polymer matrix and the nanopigments to enhance the reinforcement properties of the obtained biomaterials for other potential applications.

## Figures and Tables

**Table 1:** 2^4–1^ fractional experimental design matrix and chlorophyll dye adsorbed over the initially added dye (%).

Experiment	Nanoclay	Surfactant (wt%) *	Biomordant (wt%)	Silane (wt%)	Adsorbed Dye (Ads, %)
1	HT	5	0	5	98.13
2	HT	0	0	0	98.44
3	HT	5	1	0	97.97
4	HT	0	1	5	98.12
5	MMT	5	0	0	97.83
6	MMT	0	0	5	97.52
7	MMT	0	1	0	95.66
8	MMT	5	1	5	96.62

* Cetylpyridinium bromide (CPB) was used for montmorillonite (MMT) and sodium dodecyl sulfate (SDS) for hydrotalcite (HT).

**Table 2:** Variance analysis for the synthesis of chlorophyll hybrid nanopigments.

Factor	Sum of Squares	DF	Mean Square	F-Value	*p*-Value
B (Surfactant)	0.2387	1	0.2387	216.15	0.0432
C (Mordant)	2.8227	1	2.8227	2555.63	0.0126
D (Silane)	1.8126	1	1.8126	1641.11	0.0157
AB (Nanoclay-Surfactant)	0.8489	1	0.8489	768.59	0.0230
AC (Nanoclay-Mordant)	0.1624	1	0.1624	147.08	0.0524
AD (Nanoclay-Silane)	0.2549	1	0.2549	230.78	0.0418
Total Error	0.0011	1	0.0011		
Total (corr.)	6.1414	7			
R^2^ (%): 99.98					
Adj R^2^ (%): 99.87					

**Table 3:** CIELAB parameters for INZEA-based bionanocomposites containing 7 wt% of unmodified MMT and HT and chlorophyll hybrid nanopigments (m ± SD; n = 3).

Formulations	L*	a*	b*	Gloss	∆E*
White Control	99.47 ± 0.00	−0.08 ± 0.01	−0.08 ± 0.01	121	-
INZEA	81.66 ± 0.32	0.65 ± 0.05	5.11 ± 0.04	68 ± 3	18.56 ± 0.30
INZEA_MMT	79.50 ± 0.74	0.90 ± 0.07	8.83 ± 0.16	60 ± 5	21.89 ± 0.66
INZEA_MMT-C	74.22 ± 0.23	0.33 ± 0.04	12.00 ± 0.14	64 ± 1	28.00 ± 0.16
INZEA_MMT-C_2	68.29 ± 0.05	0.13 ± 0.02	12.30 ± 0.03	70 ± 1	33.55 ± 0.04
INZEA_HT	86.22 ± 0.19	0.14 ± 0.04	4.22 ± 0.08	57 ± 1	13.93 ± 0.19
INZEA_HT-C	74.67 ± 0.24	−0.07 ± 0.01	18.71 ± 0.15	65 ± 1	31.12 ± 0.10
